# The ever ongoing cosmetic quest to change eye colour

**DOI:** 10.1111/aos.16798

**Published:** 2024-11-15

**Authors:** Richard H. C. Zegers

**Affiliations:** ^1^ Department of Ophthalmology Amsterdam UMC, AMC Amsterdam The Netherlands; ^2^ Oogkliniek Maastricht Maastricht The Netherlands

**Keywords:** eye colour, eye drops, implant, keratopigmentation, laser, tattoo

## Abstract

**Purpose:**

To provide a historical overview of the various efforts to change eye colour for purely cosmetic reasons, along with the associated potential risks and harms.

**Methods:**

Literature and online search.

**Results:**

Eye drops containing adrenaline were used during World War II on involuntary inmates of concentration camp Auschwitz‐Birkenau. Prostaglandin drops, used in glaucoma therapy since 1996, can increase iris pigmentation as an undesired side effect. Commercial drops, available since 2011, are not effective and potentially dangerous. Iris implants, commercially available since 2011, led to serious complications like corneal decompensation, uveitis and glaucoma, and for this reason, implants had to be removed, leaving some patients almost blind. Also commercially in use since 2011 are laser treatments to make brown eyes blue. Among the possible complications are anterior uveitis and (pigmentary) glaucoma. Corneal tattooing has existed for almost 2000 years. Complications of modern, intrastromal keratopigmentation include corneal perforation, bacterial infection, allergic or toxic reaction to pigment, migration of pigment, and functional complications like visual field limitation and light sensitivity. Personal identity and self‐esteem are likely contributing factors to undergo this potentially harmful cosmetic eye surgery. In addition to the earlier discussed complications, the artificial layer of colour can obscure ocular pathology of the cornea or iris. As the majority of individuals undergoing these procedures are relatively young, problems may arise in the future when they will need cataract or other eye surgeries.

**Conclusion:**

Permanently changing eye colour in healthy eyes for purely cosmetic reasons is a risky procedure.

## 1 INTRODUCTION

Changing eye colour has been practiced for almost 2000 years. Especially in the last two decades, there seems to be an increased public interest in altering the eye colour permanently, as evidenced by the substantial growth in the number of commercial providers in this business. Although this practice initially aimed to disguise disfigured eyes caused by (congenital) disease or trauma and to improve visual function, it recently also serves purely cosmetic purposes. Despite the dubious results and serious health risks associated with this procedure, the quest to find a gold standard for altering eye colour continues to this day. This article provides a historical overview of the various efforts to change eye colour for purely cosmetic reasons and the associated potential risks and harms.

## 2 METHODS

A literature search was conducted using PubMed, Google Scholar, and various internet sources, employing combinations of one or more of the following keywords: “eye colour,” “change,” “alter,” “methods,” “adverse effects”, “risks”, “complications,” “eye drops,” “surgery,” “laser,” “tattoo,” “keratopigmentation”, and “pigment”. Additionally, any relevant hits containing names of brands, companies and individuals were used to conduct further searches on the internet.

ChatGTP‐4 was utilized to verify (historical) information and for editing purposes, but was not relied upon as the primary search engine and definitely not used for generation of content.

## 3 RESULTS

### 3.1 Eye drops I: Nazi experimentation

Although the exact motivation is not known, in 1943 and 1944 the German government ordered the Kaiser Wilhelm Institute (KWI) in Berlin to perform scientific research on the phenogenetics of eye colour (Hesse, 2001). The director of the KWI, geneticist Otmar von Verschuer, assigned this so‐called “Project Eye Colour” (Projekt Augenfarbe) to his employee Karin Magnussen, a fanatic Nazi. In turn, Magnussen collaborated with notorious Nazi physician Josef Mengele. Mengele, who was stationed at the Auschwitz‐Birkenau concentration camp, administered adrenaline drops into the eyes of inmates, mainly children, on Magnussen's directive (Zegers, 2020). The goal was to change eye colour. Among the involuntary victims was a Sinti family that showed a high prevalence of heterochromia iridis (Hesse, 2001; Zegers, 2020). The family members were killed, their eyes enucleated, and sent to the KWI for further examination. The adrenaline had no influence on eye colour, except that eyes could appear darker due to pupillary dilation, one of the sympathetic effects of adrenaline. Nevertheless, due to the long‐term use of adrenaline as a chronic anti‐glaucoma therapy after World War II, we now know that it can sometimes cause hyperpigmentation of the conjunctiva and, very rarely, of the cornea, but not of the iris (Domarus, 1977). The systemic effects of adrenaline, particularly severe hypertension, would have been potentially dangerous, and it resulted at least in the death of a neonate prisoner (Zegers, 2020). Magnussen tried to publish her findings from this medical experiment several years after the war, but never succeeded, as reviewers suspected the materials came from prisoners of concentration camps (Hesse, 2001).

### 3.2 Eye drops II: Undesired side effect

Prostaglandin eye drops are used worldwide in the treatment of glaucoma as intraocular pressure‐lowering agents since 1996. The most frequently prescribed prostaglandin analogues are latanoprost, travoprost, bimatoprost, and the less widely used unoprostone, all of which have darkening of the iris as a potential side effect (Holló, 2007). Although this effect is more pronounced in light‐coloured eyes, brown eyes can also become darker (Hara, 2001). The mechanism of action involves an increase in melanin pigment granules in the anterior border layer and stroma of the iris, as well as a thickening of the anterior border layer of the iris (Albert et al., 2008). Older persons seem to be more susceptible to this side effect than younger ones (Arranz‐Marquez & Teus, 2007). The same applies to different ethnic groups: Japanese eyes are reported to have an increase in iris pigmentation of approximately 50%, higher than reported for people of European descent (Latanoprost‐Induced Iris Pigmentation Study Group, 2006). To date, no commercial prostaglandin‐based preparations specifically for altering eye colour are available, but this may only be a matter of time. This is exactly what happened with another, often desired side effect of prostaglandin analogues: the growth of eyelid lashes. For this purpose, a commercial, U.S. Food and Drug Administration (FDA)‐approved bimatoprost‐based solution became available in 2008 (U.S. Food & Drug Administration n.d.). It has, however, not been approved for use in Europe.

### 3.3 Eye drops III: Commercial preparations

Eye drops claiming to alter eye colour have been available, mainly online, since 2011 with names like iColour (also available as a balm for periorbital application), Iris Ink and Crystal Drops. Theoretically, eye colour would become lighter due to one of the ingredients, n‐acetylglucosamine, which has the ability to inhibit melanin production. This property has been demonstrated to be effective in hyperpigmentation of the skin, but has not yet been scientifically established in the melanocytes of the iris (Bissett et al., 2007). Hence, a possible working mechanism and safety profile of these products are unknown, let alone that an effect has been demonstrated to alter eye colour. Moreover, these eye drops are not approved by major regulatory bodies like the Conformité Européenne (CE) or FDA. Given their illegal status, microbial contamination of the contents and/or bottles is also a source of concern. There are no scientific papers addressing these kinds of eye drops, but depending on the exact ingredients, one can expect inflammatory and allergic reactions in the anterior segment of the eye. Another potentially dangerous side effect could be that the retinal pigment is targeted by the n‐acetylglucosamine. This could seriously compromise visual function, as retinal pigment epithelium plays a key role in, among several other functions, absorption of light, maintenance of the blood‐retina barrier and vitamin A metabolism.

### 3.4 Iris implants

**FIGURE 1:**
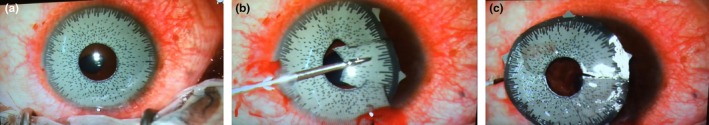
Surgical explantation of an BrightOcular iris implant used for cosmetic reasons. (a) At the start of the operation. (b) During the procedure, two radial incisions are made and then the implant is extracted through a corneal tunnel. (c) The explanted implant. Courtesy of Marc Muraine, MD, PhD, Rouen, France.

Iris implants can be used in patients with incomplete or absent irises, caused by aniridia, disease or trauma, and improve cosmetic appearance as well as visual function. The first therapeutic iris prosthesis was implanted by American ophthalmologist Peter Choyce in 1956 (Pandey & Apple, 2005). In 2006, a patent application was granted for an artificial iris implant, insertable into the anterior chamber to cover (parts of) a natural iris for cosmetic and/or medical reasons: the NewColorIris implant (Google Patents, 2006). The flap portions of this silicone implant could be placed into the angle recess of the anterior chamber to keep the implant in position. The implant never received approval from the major regulatory bodies. Thanks to highly polished commercials (like https://www.youtube.com/watch?v=OCCrHEcN33Y&t=3s), people all over the world travelled to Panama City, Panama, the only place where the NewColorIris could be implanted. It was not long thereafter that the first reports describing serious complications were published, and for this reason, implants had to be removed, leaving some patients almost blind (Hoguet et al., 2012; Sikder et al., 2011; Thiagalingam et al., 2008). Complications included endothelial cell loss, leading to corneal decompensation, and uveitis and glaucoma, both potentially blinding conditions. NewColorIris disappeared from the market and was followed by BrightOcular in 2012 (Figure 1a) (Google Patents, 2012). Made of inert material; this prosthesis contains arc sections that minimize iris contact and allow better passage for aqueous humour. For BrightOcular, no CE‐marking or FDA approval has been granted yet. Despite claims that this implant is safer than its predecessor, the same types of ocular complications, including the need for explantation, were reported (Figure 1b,c) (El Chehab et al., 2020; Galvis et al., 2016; Mansour et al., 2016). Nevertheless, on the website of BrightOcular, several testimonials from happy clients can still be found (https://brightocular.com/videos/). Currently, the procedure is available in a limited number of countries, including India, Turkey, and Columbia.

### 3.5 Laser depigmentation

In 2011, news broke that brown eyes could be turned blue by means of a laser treatment, but in scientific literature, this claim could not be found (Freeman, 2011). Nevertheless, exactly a decade later, the Q‐switched crystal Nd: laser at double frequency (532 nm) has been claimed to be the most promising treatment for cosmetic iris depigmentation; however, this has to be confirmed by other studies (Grimaldos Ruiz, 2021). The procedure can also be performed with a ‘standard’ neodymium: yttrium–aluminium–garnet (Nd: YAG laser) (D'Oria et al., 2022). Other, unspecified and hence not CE‐marked or FDA‐approved laser treatments, are, for example, offered by South‐America based companies, having names like Yeux Clairs (https://www.yeuxclairs.com/), NewEyes (https://eyecos.eu/en/neweyes‐laser‐7g/#), and Stroma (https://www.stromamedical.com/).

**FIGURE 2:**
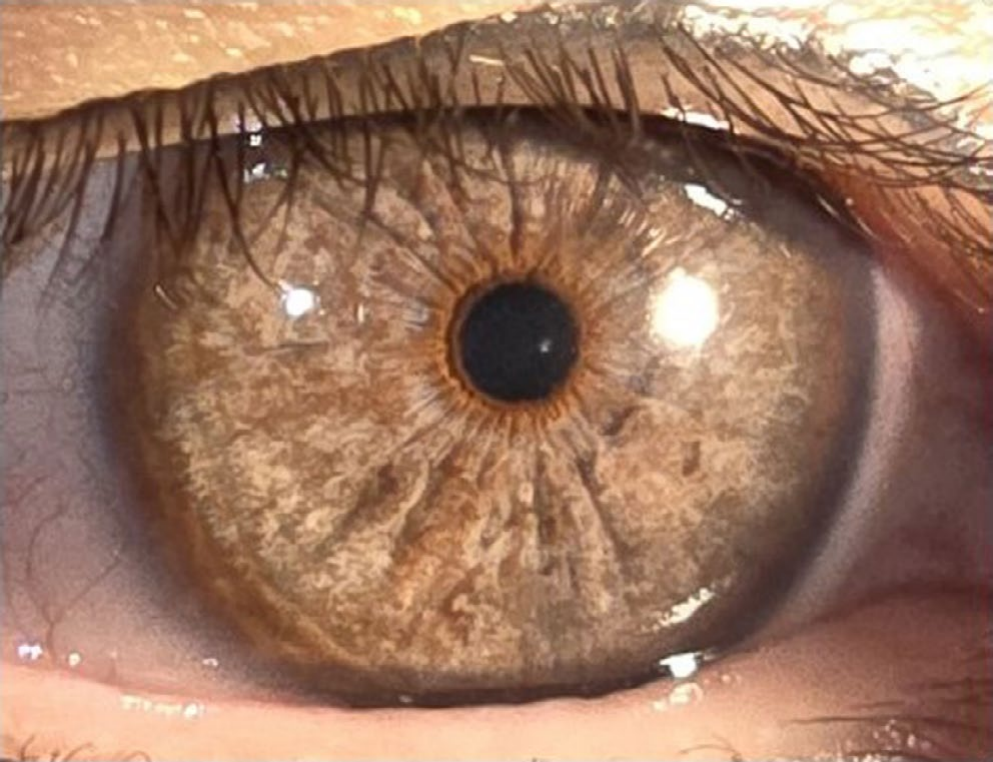
Laser depigmentation. The irregular iris surface of an eye that underwent laser treatment for depigmentation to achieve a lighter appearance. Courtesy of Georges Baïkoff, MD, Nice, France.

All applications have in common that they target the melanin inside the iris, have a photoablative effect, and generate miniparticles (Figure 2). Among the possible serious complications are anterior uveitis and (pigmentary) glaucoma; of the latter several dramatic examples can be found in the medical literature, in which pigmentary glaucoma needed surgery and permanent bilateral visual field damage was the result (Liu et al., 2023; Ong et al., 2021; Swampillai et al., 2023).

### 3.6 Keratopigmentation

**FIGURE 3:**
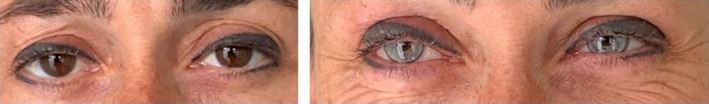
Femtosecond laser‐assisted keratopigmentation. Left: Preoperative view of the brown‐coloured iris. Right: Postoperative view of the green‐coloured cornea. Courtesy of Georges Baïkoff, MD, Nice, France.

Keratopigmentation, or corneal tattooing, is the oldest technique to alter eye colour and has existed for almost 2000 years. The Greco‐Roman physician Aelius Galenus, better known as Galen of Pergamon, is credited with performing the first corneal tattoo in AD 150, intended to camouflage a corneal opacity (Doganay et al., 2020). After cauterizing the corneal surface, dye containing pigments like powdered nutgalls, iron, or copper sulphate was applied to the eye. In 1869, French oculoplastic surgeon Louis Von Wecker introduced a new method by applying China ink over the cornea and subsequently puncturing it into the cornea with a grooved needle (Roy, 1938). This marked the beginning of modern keratopigmentation. Corneal tattooing can be indicated to reduce photophobia and glare in patients with aniridia, albinism, and other conditions that cause light sensitivity, and to improve visual function in cases of iris defects or refractory diplopia. In particular, over the last decade, attention has been drawn to the application of keratopigmentation to change eye colour in healthy eyes (Figure 3) (Alió et al., 2016).

Several techniques can be used for keratopigmentation. Based on Von Wecker's invention and more or less similar to traditional tattooing techniques, pigments can be impregnated into the superficial layers of the cornea by means of needle puncture. This technique can be performed manually or automatically, with special devices available for this purpose. Complications associated with the superficial technique include pigment non‐homogeneity, colour fading, intraoperative perforation, uveitis and recurrent corneal erosions (Hasani et al., 2020).

**FIGURE 4:**
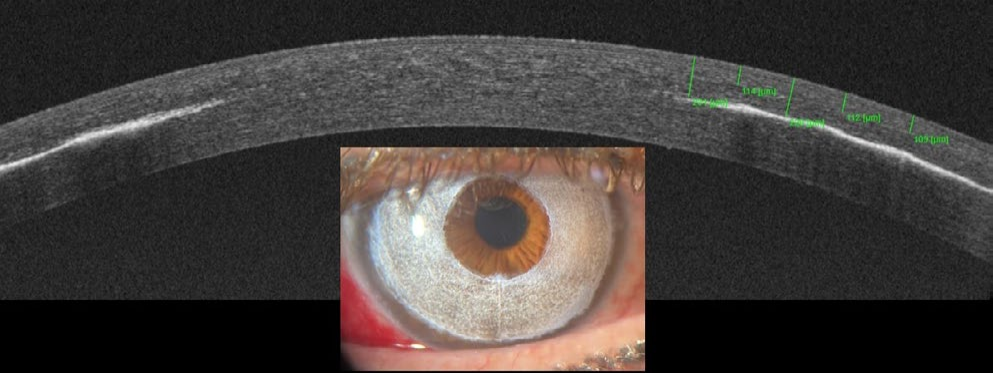
Femtosecond laser‐assisted keratopigmentation. Pigments are injected into a corneal tunnel created with a femtosecond laser at a depth of 200 μm. Courtesy of Georges Baïkoff, MD, Nice, France.

In modern techniques, pigment dye is applied intrastromally (Figure 4). The current state‐of‐the‐art method involves using a femtosecond laser to create a circular intrastromal pocket, first described for cosmetic reasons in medical literature in 2015 (Ferrari & Morin, 2015). A single‐tunnel or double‐tunnel technique can be used (D'Oria et al., 2022). During this procedure, the pupil diameter is set to 5.5 mm and the outer diameter to 9.5 mm, which can optionally be further dissected to the limbus. Via a superior opening, pigment is injected into the pocket or pockets. This procedure first became available in Spain (https://www.bluegreenmedical.com/) and later in other countries including France (https://docteur‐ferrari.com/en/) and the USA (https://www.keratonyc.com/). When a femtosecond laser is not available, a pocket can be created manually, starting from the limbus towards the border of a previously marked pupil.

Complications of intrastromal keratopigmentation include corneal perforation (less with femtosecond laser), bacterial infection, corneal neovascularisation, allergic or toxic reaction to pigment, migration of pigment, change or fading of colour, and functional complications like visual field limitation, light sensitivity and possibly MRI alterations (Hasani et al., 2020). Pigment‐related problems, however, seem to be less prevalent with third generation mineral micronized, CE‐marked pigments (for example Biochromaeyes®; https://www.biotic.fr/en/content/27‐corneal‐tattooing) than other, previously used pigments like Indian or Chinese ink, animal uveal pigment, platinum chloride and soot.

## 4 DISCUSSION

Why do people elect to undergo potentially harmful cosmetic eye colour‐changing procedures, which can even lead to blindness in previously perfectly healthy eyes? In the case of the horrific Nazi experiment, the doomed victims were left with no choice. Nowadays, more people than ever before volunteer to have their eye colour changed and are prepared to pay over €10 000 for the procedure (https://www.keratonyc.com/frequently‐asked‐questions‐about‐eye‐color‐change). Personal identity and self‐esteem are likely contributing factors, whether dictated by social and cultural norms or enhanced by social media and influencers, of which numerous examples can be found on the internet (https://www.tiktok.com/@flaak_new_color/video/7362232675828092193). A variety of individuals have undergone these procedures, including a lawyer and a physician who received iris implants (El Chehab et al., 2020).

The explosive commercial growth in the eye colour‐changing business has its advantages: materials and techniques develop much faster than they would have in the absence of commercial interest, and subsequent innovations may also benefit patients in need of eye colour changes for medical reasons. In line with this, the very first international congress fully dedicated to eye colour changing was held this year in Spain (https://www.kolor‐congress.org/).

Unlike surgical alternatives, the market for eye drops appears to have remained stable for the last decade, with little indication of imminent change. The currently available eye drops, however, have not been proven effective and carry potential health risks.

In addition to the earlier discussed variety of complications, further concerns arise with penetrating procedures. What this kind of surgery has in common is that ocular pathology, such as corneal or iris disease, can be obscured by the artificial layer of colour, potentially going unnoticed by both the patient and the ophthalmologist. This oversight may have serious and even lethal consequences, such as when an iris melanoma is missed in its early stages.

Moreover, the vast majority of individuals undergoing purely cosmetic eye colour‐changing procedures are relatively young. One study found an average age of 34.2 (± 10.9) years and in another, large series, the average age was 33.7 (± 9.68) years (El Chehab et al., 2020; Grimaldos Ruiz, 2021). As this generation ages and develops cataracts requiring surgery, the procedure may become more challenging and carry a higher risk of complications. The same applies to other ophthalmological operations that may be indicated later in life.

Lastly, most eye colour‐changing materials and techniques are relatively new, so the long‐term adverse effects or complications that may arise are not yet fully understood.

## 5 CONCLUSION

Permanently changing eye colour in healthy eyes for cosmetic reasons is a risky intervention. Apart from many potentially serious medical complications associated with different procedures, in case of penetrating surgery difficulties may also arise in the long run, such as missed diagnoses of ocular pathology, or an increased risk of complications during necessary eye surgery. All these issues can lead to irreversible damage, deterioration of visual function, loss of the eye, and in extreme cases, may even be life‐threatening.

## FUNDING INFORMATION

None declared.
